# Shared transcription factors contribute to distinct cell fates

**DOI:** 10.4161/21541264.2014.978173

**Published:** 2015-01-06

**Authors:** Felicia SL Ng, Fernando J Calero-Nieto, Berthold Göttgens

**Affiliations:** Department of Haematology; Wellcome Trust and MRC Cambridge Stem Cell Institute & Cambridge Institute for Medical Research; Cambridge University; Cambridge, UK

**Keywords:** binding motif, hematopoiesis, mast cell, regression model

## Abstract

Genome-wide transcription factor (TF) binding profiles differ dramatically between cell types. However, not much is known about the relationship between cell-type-specific binding patterns and gene expression. A recent study demonstrated how the same TFs can have functional roles when binding to largely non-overlapping genomic regions in hematopoietic progenitor and mast cells. Cell-type specific binding profiles of shared TFs are therefore not merely the consequence of opportunistic and functionally irrelevant binding to accessible chromatin, but instead have the potential to make meaningful contributions to cell-type specific transcriptional programs.

## Abbreviations

TF: transcription factor
ChIP-seq: ChIP sequencing
RNA-seq: RNA sequencing
GAM: generalized additive model

## 

Transcription factors (TFs) are important regulators of cell-type-specific gene expression, and represent the paradigm for DNA-binding proteins that influence cellular development. Hematopoiesis has long served as a model to study the transcriptional control of cell type specification, and transcriptional regulatory elements for several major regulators of hematopoietic stem cells (HSCs) have been studied in detail using both mammalian and lower vertebrate model organisms.^1-14^ Nevertheless, much remains to be learned about the cell-type-specific transcriptional mechanisms that govern hematopoietic cell type identity. A thorough investigation into how TFs can contribute to distinct transcriptional programs, therefore, is critical for understanding how cells acquire and maintain their identity. During hematopoietic stem cell differentiation and blood cell development, it is well known that these cells share many TFs across distinct lineages. The PU.1 TF, for example, auto-regulates itself in 2 different cell types (myeloid and B-cells) through co-operative interaction with distinct cell-specific TFs.^6^ Furthermore, the SCL TF is required not only for specifications of HSC but also differentiation along the erythroid and megakaryocytic lineages.^15,16^ Interestingly, binding sites for the same TF in 2 cell types have been shown to be largely non-overlapping and behave in a cell-type-specific manner.^17,18^ These findings, therefore, raise the question as to whether both cell-type-specific and common TF binding patterns in the genome have functional consequences for defining cell fate. Several recent review papers provided in-depth discussions on what may constitute functional TF binding, suggestions to discover functional enhancers or indeed if co-operative TF binding represents a continuum rather than just 2 states.^19-22^ These discussions revealed many unresolved questions on the topic including the fundamental need to identify binding activity that results in stable cellular states.

In a recent study, we examined the nature of binding site preferences and co-occupancy in 2 closely related cell types.^23^ The cell types compared were primary mast cells and a multipotent hematopoietic progenitor cell line, HPC7,^24^ which we have established as a useful model for studying early blood stem/progenitor cells.^25,26^ Gene expression profiling by RNA-sequencing (RNA-seq) revealed many significant differences in the expression profiles of the 2 cell types. Nevertheless, many known TFs display similar expression in both cell types and this includes key regulators of hematopoietic stem cells (i.e., E2A, Erg, Fli1, Gata2, Lmo2, Meis1, PU.1, Runx1, and Scl). Generation of genome-wide TF binding maps by chromatin-immunoprecipitation followed by sequencing (ChIP-seq) of those ‘shared’ TFs uncovered largely non-overlapping promoter and enhancer occupancy between the 2 cell types. For most of these HSC TFs, it was not known whether (and if so, how) they might have a role in the transcriptional regulation of mast cells.Having established a unique large-scale dataset for comparative analysis, we next asked the question as to what is the role of ‘shared’ TFs in controlling mast cell specific transcriptional programs?

**Figure 1.**
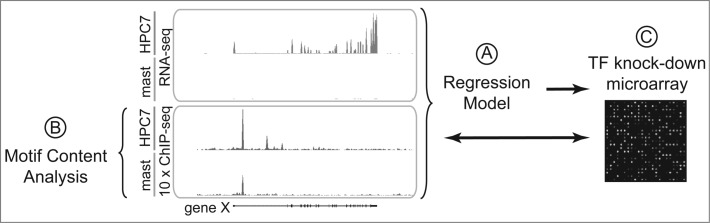
Summary of computational and experimental approaches in the genome-wide comparison of HPC7 blood progenitor and mast cells. (**A**) Regression models using differential TF occupancy and differential gene expression. (**B**) Motif content analysis of cell-type-specific and common binding regions. (**C**) shRNA perturbation experiments compared to TF binding and gene expression data.

We examined the observation of distinct binding patterns further by quantifying the differences in binding and their relationship to gene expression in a regression model (**Fig. 1A**). Regression models provide a useful and simple approach to quantify the relationships between multiple predictor variables (i.e., ‘shared’ TFs) to a response variable (i.e., gene expression). Furthermore, the availability of high resolution genome-wide data (i.e., ChIP-seq and RNA-seq) allowed the construction of accurate predictive models. By considering genes bound by at least one of these TFs, these models describe gene expression as a function of combinatorial effects of one or more relative TF binding strengths. In the past, other studies have also utilized regression statistics to build a variety of prediction models that include, for example, TF binding data, histone modification, and consensus binding motifs.^27,28^ Until recently, these studies have focused on predicting gene expression in one cell type, obtaining high levels of correlation with observed data. However, applying the model to another cell type often results in poor accuracy since static expression levels were used to construct the model. Our study, on the other hand, employed regression models for 2 cell types to predict changes in gene expression. Thus, when differential promoter and distal enhancer occupancy were encoded into the model, quantitative changes in TF binding were found to be predictive of quantitative changes in differential gene expression. Moreover, prediction accuracy improved when multiple binding events were taken into account.

**Figure 2.**
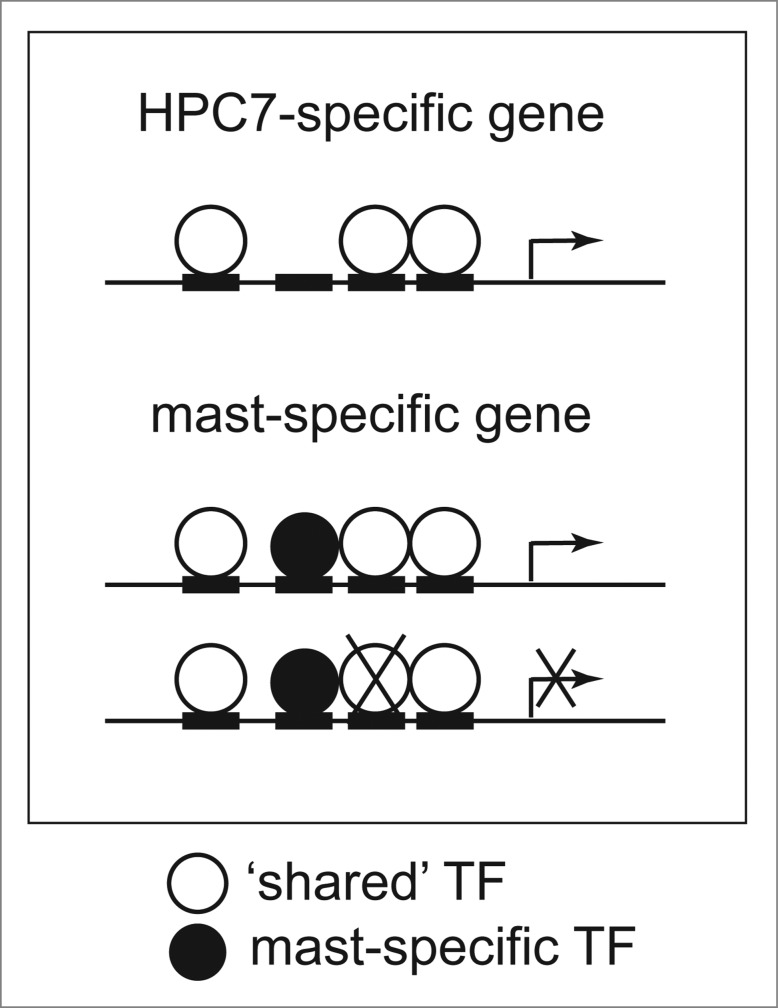
Schematic of gene regulation by ‘shared’ and mast-specific TFs. Both mast-specific and ‘shared’ TFs are important for cell-type-specific gene regulation. Knocking-down a ‘shared’ TF in mast cells results in significant changes to gene expression for a large number of mast-specific genes.

Many regression models described so far assumed that TF binding and gene expression have a linear relationship. Although linearity provides an easy means for performing computations, it is known that this relationship could be non-linear, at least for a subset of TFs. Indeed, gene expression has been shown to be a non-linear function of TF binding as shown in an analysis of K562 and GM112878 cell lines.^29^ Here, the authors used generalized additive models^30,31^ (GAMs) to identify TFs that influence cell-type-specific gene expression. Similarly, in the HPC7 and mast data set, using GAMs to predict changes in differential gene expression improved prediction accuracy compared to linear regression in 2 ways: (i) allowing non-linearity of TF binding and; (ii) incorporating TF interaction terms to account for co-operative interaction between 2 TFs. Importantly, we could conclude from our modeling that ‘shared’ TFs play an important role in HPC7 and mast cell transcriptional programs, since their differential binding is predictive of expression.

TF binding depends on interactions with DNA and other TFs at regulatory regions, although it has been suggested that in some occasions it can be largely driven by its cellular environment with no functional consequences. We compared the motif content of common and cell-type-specific regulatory regions (**Fig. 1B**) under the hypothesis that the presence of binding motifs for ‘shared’ TF at cell-type-specific bound regulatory regions may provide additional evidence of direct involvement of the ‘shared’ TFs in cell-type-specific programs. We uncovered large numbers of cell-specific and common binding regions that contained consensus sequence motifs for the ‘shared’ TFs. To further analyze the role of ‘shared’ TFs in cell-type-specific genetic programs, we followed 2 approaches: (i) we reduced the levels of some of the ‘shared’ TFs (E2A, Erg, Fli1, Gata2, Lmo2, PU.1) by performing shRNA perturbation experiments (**Fig. 1C**) and analyzed the effect on cell-type-specific genetic programs and; (ii) we mutated the putative binding motifs present in cell-type-specific promoters and analyzed the direct effect on gene expression. Our first approach showed that individual knock-down of these regulators significantly affects large numbers of regulated targets with significant changes in cell-type-specific gene expression. Worthy of note, we also found that reduction of the levels of one ‘shared’ factor can affect the recruitment of other ‘shared’ factors. Our second approach demonstrated that ablation of binding motifs for ‘shared’ TFs resulted in strong reduction or even complete abolition of promoter activity.

Although binding of ‘shared’ TFs without functional consequences cannot be ruled out for a given specific binding event, our results clearly indicate that the same TF can play an active and determinant role in different cell-type-specific transcriptional programs.

## Conclusions and Future Directions

Our recent analysis of genome-wide binding sites and gene expression in HPC7 and mast cells has provided new insights into our understanding of lineage-specific transcriptional regulation during differentiation. Furthermore, it reports comprehensive genome-scale data for primary mast cells, where until now very little genome-wide data existed. The use of computational and experimental approaches provided several lines of evidence to show that occupancy of distinct regions in different cell lineages by the same TFs are functionally important and not just a consequence of the cellular environment (**Fig. 2**).

It is well known that combinatorial TF binding is prevalent in the regulation of metazoan gene expression. However, the specific rules governing these TF interactions and the effects of individual regulatory elements on gene expression remain largely unknown, even though they are recognized to have broad implications for cellular reprogramming. In a study of differentiating embryonic stem cells, for example, the Isl1 protein functions as a component of 2 different regulatory complexes that differ by one TF but lead to either spinal or cranial motor neuron formation.^32^ TF binding site arrangements, genomic sequence context (e.g., flanking bases, GC content), enhancer composition (heterotypic or homotypic arrangements of TF binding), and DNA tertiary structure are all expected to influence transcriptional activity. Elucidation of the molecular mechanisms, therefore, will improve our understanding of how the same TF contributes to distinct cell fates. Research in this area has so far provided little consensus and one example is demonstrated by the ‘enhanceosome’ vs. ‘TF collective’ debate. The classical example of TF complex formation as proposed by the enhanceosome model requires a precise configuration of multiple TFs to function as a unit of regulation. In the well-studied *IFN-*β enhancer, synergistic interaction between all essential TFs is necessary and leads to an ‘all-or-nothing’ response.^33^ At the opposite end of the spectrum, the ‘TF collective’ model suggests that the effects of TF binding on gene expression are cumulative with varying degrees of potency and redundancy. A recent study comparing TF binding in 2 mouse strains has shown that both ‘shared’ and cell-type-specific TFs are important for establishing the epigenetic and transcriptomic landscapes in mouse macrophages.^34^ Of note, comparison of naturally occurring single nucleotide polymorphisms (SNPs) that differed between the 2 mouse strains revealed strain-specific binding site motifs that correlated with strain-specific gain or loss of TF binding and influenced the recruitment of cell-type-specific factors. The findings in this study also emphasized the presence of binding sites and nucleosome conformation as important features for co-operative TF binding although defined distances between TFs was not crucial. Integrative analyses taking into account dynamic binding, sequence information and 3-dimensional DNA structure to infer general principles of transcriptional regulation would, therefore, help to resolve the disparate findings in this field.

## References

[1] Landry JR, Kinston S, Knezevic K, Donaldson IJ, Green AR, Gottgens B. Fli1, Elf1, and Ets1 regulate the proximal promoter of the LMO2 gene in endothelial cells. Blood 2005; 106:2680-7; PMID:15994290; http://dx.doi.org/10.1182/blood-2004-12-4755 15994290

[2] Bockamp EO, Fordham JL, Gottgens B, Murrell AM, Sanchez MJ, Green AR. Transcriptional regulation of the stem cell leukemia gene by PU.1 and Elf-1. J Biol Chem 1998; 273:29032-42; PMID:9786909; http://dx.doi.org/10.1074/jbc.273.44.29032 9786909

[3] Delabesse E, Ogilvy S, Chapman MA, Piltz SG, Gottgens B, Green AR. Transcriptional regulation of the SCL locus: identification of an enhancer that targets the primitive erythroid lineage in vivo. Mol Cell Biol 2005; 25:5215-25; PMID:15923636; http://dx.doi.org/10.1128/MCB.25.12.5215-5225.2005 15923636 PMC1140604

[4] Barton LM, Gottgens B, Gering M, Gilbert JG, Grafham D, Rogers J, Bentley D, Patient R, Green AR. Regulation of the stem cell leukemia (SCL) gene: a tale of two fishes. Proc Natl Acad Sci U S A 2001; 98:6747-52; PMID:11381108 11381108 10.1073/pnas.101532998 PMC34424

[5] Pimanda JE, Chan WY, Donaldson IJ, Bowen M, Green AR, Gottgens B. Endoglin expression in the endothelium is regulated by Fli-1, Erg, and Elf-1 acting on the promoter and a -8-kb enhancer. Blood 2006; 107:4737-45; PMID:16484587; http://dx.doi.org/10.1182/blood-2005-12-4929 16484587

[6] Leddin M, Perrod C, Hoogenkamp M, Ghani S, Assi S, Heinz S, Wilson NK, Follows G, Schonheit J, Vockentanz L, et al. Two distinct auto-regulatory loops operate at the PU.1 locus in B cells and myeloid cells. Blood 2011; 117:2827-38; PMID:21239694; http://dx.doi.org/10.1182/blood-2010-08-302976 21239694 PMC3062295

[7] Bee T, Ashley EL, Bickley SR, Jarratt A, Li PS, Sloane-Stanley J, Gottgens B, de Bruijn MF. The mouse Runx1 +23 hematopoietic stem cell enhancer confers hematopoietic specificity to both Runx1 promoters. Blood 2009; 113:5121-4; PMID:19321859; http://dx.doi.org/10.1182/blood-2008-12-193003 19321859

[8] Wilson NK, Timms RT, Kinston SJ, Cheng YH, Oram SH, Landry JR, Mullender J, Ottersbach K, Gottgens B. Gfi1 expression is controlled by five distinct regulatory regions spread over 100 kilobases, with Scl/Tal1, Gata2, PU.1, Erg, Meis1, and Runx1 acting as upstream regulators in early hematopoietic cells. Mol Cell Biol 2010; 30:3853-63; PMID:20516218; http://dx.doi.org/10.1128/MCB.00032-10 20516218 PMC2916401

[9] Gottgens B, Ferreira R, Sanchez MJ, Ishibashi S, Li J, Spensberger D, Lefevre P, Ottersbach K, Chapman M, Kinston S, et al. cis-Regulatory remodeling of the SCL locus during vertebrate evolution. Mol Cell Biol 2010; 30:5741-51; PMID:20956563; http://dx.doi.org/10.1128/MCB.00870-10 20956563 PMC3004278

[10] Nottingham WT, Jarratt A, Burgess M, Speck CL, Cheng JF, Prabhakar S, Rubin EM, Li PS, Sloane-Stanley J, Kong ASJ, et al. Runx1-mediated hematopoietic stem-cell emergence is controlled by a Gata/Ets/SCL^-^regulated enhancer. Blood 2007; 110:4188-97; PMID:17823307; http://dx.doi.org/10.1182/blood-2007-07-100883 17823307 PMC2234795

[11] Gottgens B, Nastos A, Kinston S, Piltz S, Delabesse EC, Stanley M, Sanchez MJ, Ciau-Uitz A, Patient R, Green AR. Establishing the transcriptional programme for blood: the SCL stem cell enhancer is regulated by a multiprotein complex containing Ets and GATA factors. EMBO J 2002; 21:3039-50; PMID:12065417; http://dx.doi.org/10.1093/emboj/cdf286 12065417 PMC126046

[12] Levantini E, Lee S, Radomska HS, Hetherington CJ, Alberich-Jorda M, Amabile G, Zhang P, Gonzalez DA, Zhang J, Basseres DS, et al. RUNX1 regulates the CD34 gene in haematopoietic stem cells by mediating interactions with a distal regulatory element. EMBO J 2011; 30:4059-70; PMID:21873977; http://dx.doi.org/10.1038/emboj.2011.285 21873977 PMC3209778

[13] Chan WY, Follows GA, Lacaud G, Pimanda JE, Landry JR, Kinston S, Knezevic K, Piltz S, Donaldson IJ, Gambardella L, et al. The paralogous hematopoietic regulators Lyl1 and Scl are coregulated by Ets and GATA factors, but Lyl1 cannot rescue the early Scl^−^/− phenotype. Blood 2007; 109:1908-16; PMID:17053063; http://dx.doi.org/10.1182/blood-2006-05-023226 17053063

[14] Landry JR, Bonadies N, Kinston S, Knezevic K, Wilson NK, Oram SH, Janes M, Piltz S, Hammett M, Carter J, et al. Expression of the leukemia oncogene Lmo2 is controlled by an array of tissue-specific elements dispersed over 100 kb and bound by Tal1/Lmo2, Ets, and Gata factors. Blood 2009; 113:5783-92; PMID:19171877; http://dx.doi.org/10.1182/blood-2008-11-187757 19171877

[15] Hall MA, Curtis DJ, Metcalf D, Elefanty AG, Sourris K, Robb L, Gothert JR, Jane SM, Begley CG. The critical regulator of embryonic hematopoiesis, SCL, is vital in the adult for megakaryopoiesis, erythropoiesis, and lineage choice in CFU-S12. Proc Nat Acad Sci USA 2003; 100:992-7; PMID:12552125; http://dx.doi.org/10.1073/pnas.0237324100 12552125 PMC298714

[16] Mikkola HK, Klintman J, Yang H, Hock H, Schlaeger TM, Fujiwara Y, Orkin SH. Haematopoietic stem cells retain long-term repopulating activity and multipotency in the absence of stem-cell leukaemia SCL/tal-1 gene. Nature 2003; 421:547-51; PMID:12540851; http://dx.doi.org/10.1038/nature01345 12540851

[17] Hannah R, Joshi A, Wilson NK, Kinston S, Gottgens B. A compendium of genome-wide hematopoietic transcription factor maps supports the identification of gene regulatory control mechanisms. Exp Hematol 2011; 39:531-41; PMID:21338655; http://dx.doi.org/10.1016/j.exphem.2011.02.009 21338655

[18] Wei G, Abraham BJ, Yagi R, Jothi R, Cui K, Sharma S, Narlikar L, Northrup DL, Tang Q, Paul WE, et al. Genome-wide analyses of transcription factor GATA3-mediated gene regulation in distinct T cell types. Immunity 2011; 35:299-311; PMID:21867929; http://dx.doi.org/10.1016/j.immuni.2011.08.007 21867929 PMC3169184

[19] Kellis M, Wold B, Snyder MP, Bernstein BE, Kundaje A, Marinov GK, Ward LD, Birney E, Crawford GE, Dekker J, et al. Defining functional DNA elements in the human genome. Proc Nat Acad Sci USA 2014; 111:6131-8; PMID:24753594; http://dx.doi.org/10.1073/pnas.1318948111 24753594 PMC4035993

[20] Spivakov M. Spurious transcription factor binding: non-functional or genetically redundant? BioEssays 2014; 36:798-806; PMID:24888900; http://dx.doi.org/10.1002/bies.201400036 24888900 PMC4230394

[21] Haeussler M, Joly JS. When needles look like hay: how to find tissue-specific enhancers in model organism genomes. Dev Biol 2011; 350:239-54; PMID:21130761; http://dx.doi.org/10.1016/j.ydbio.2010.11.026 21130761

[22] DeVilbiss AW, Sanalkumar R, Johnson KD, Keles S, Bresnick EH. Hematopoietic transcriptional mechanisms: from locus-specific to genome-wide vantage points. Exp Hematol 2014; 42:618-29.; PMID:24816274; http://dx.doi.org/10.1016/j.exphem.2014.05.004 24816274 PMC4125519

[23] Calero-Nieto FJ, Ng FS, Wilson NK, Hannah R, Moignard V, Leal-Cervantes AI, Jimenez-Madrid I, Diamanti E, Wernisch L, Gottgens B. Key regulators control distinct transcriptional programmes in blood progenitor and mast cells. EMBO J 2014; 33:1212-26; PMID:24760698; http://dx.doi.org/10.1002/embj.201386825 24760698 PMC4168288

[24] Pinto do OP, Wandzioch E, Kolterud A, Carlsson L. Multipotent hematopoietic progenitor cells immortalized by Lhx2 self-renew by a cell nonautonomous mechanism. Exp Hematol 2001; 29:1019-28.; PMID:11495708; http://dx.doi.org/10.1016/S0301-472X(01)00666-X 11495708

[25] Wilson NK, Foster SD, Wang X, Knezevic K, Schutte J, Kaimakis P, Chilarska PM, Kinston S, Ouwehand WH, Dzierzak E, et al. Combinatorial transcriptional control in blood stem/progenitor cells: genome-wide analysis of ten major transcriptional regulators. Cell Stem Cell 2010; 7:532-44; PMID:20887958; http://dx.doi.org/10.1016/j.stem.2010.07.016 20887958

[26] Wilson NK, Miranda-Saavedra D, Kinston S, Bonadies N, Foster SD, Calero-Nieto F, Dawson MA, Donaldson IJ, Dumon S, Frampton J, et al. The transcriptional program controlled by the stem cell leukemia gene Scl/Tal1 during early embryonic hematopoietic development. Blood 2009; 113:5456-65; PMID:19346495; http://dx.doi.org/10.1182/blood-2009-01-200048 19346495

[27] Conlon EM, Liu XS, Lieb JD, Liu JS. Integrating regulatory motif discovery and genome-wide expression analysis. Proc Natl Acad Sci U S A 2003; 100:3339-44; PMID:12626739; http://dx.doi.org/10.1073/pnas.0630591100 12626739 PMC152294

[28] Karlic R, Chung HR, Lasserre J, Vlahovicek K, Vingron M. Histone modification levels are predictive for gene expression. Proc Natl Acad Sci U S A 2010; 107:2926-31; PMID:20133639; http://dx.doi.org/10.1073/pnas.0909344107 20133639 PMC2814872

[29] Wang D, Rendon A, Ouwehand W, Wernisch L. Transcription factor co-localization patterns affect human cell type-specific gene expression. BMC Genomics 2012; 13:263; PMID:22721266; http://dx.doi.org/10.1186/1471-2164-13-263 22721266 PMC3441573

[30] Hastie T, Tibshirani R Generalized Additive Models. Stat Sci 1986; 1:297-310; http://dx.doi.org/10.1214/ss/1177013604

[31] Wood SN Fast stable restricted maximum likelihood and marginal likelihood estimation of semiparametric generalized linear models. J R Stat Soc B 2011; 73:3-36; http://dx.doi.org/10.1111/j.1467-9868.2010.00749.x

[32] Mazzoni EO, Mahony S, Closser M, Morrison CA, Nedelec S, Williams DJ, An D, Gifford DK, Wichterle H. Synergistic binding of transcription factors to cell-specific enhancers programs motor neuron identity. Nat Neurosci 2013; 16:1219-27; PMID:23872598; http://dx.doi.org/10.1038/nn.3467 23872598 PMC3820498

[33] Maniatis T, Falvo JV, Kim TH, Kim TK, Lin CH, Parekh BS, Wathelet MG. Structure and function of the interferon-beta enhanceosome. Cold Spring Harb Symp Quant Biol 1998; 63:609-20; PMID:10384326; http://dx.doi.org/10.1101/sqb.1998.63.609 10384326

[34] Heinz S, Romanoski CE, Benner C, Allison KA, Kaikkonen MU, Orozco LD, Glass CK. Effect of natural genetic variation on enhancer selection and function. Nature 2013; 503:487-92; PMID:24121437; http://dx.doi.org/10.1038/nature12615 24121437 PMC3994126

